# Intrasession repeatability of ocular anatomical measurements obtained with a multidiagnostic device in healthy eyes

**DOI:** 10.1186/s12886-017-0589-x

**Published:** 2017-10-18

**Authors:** David P. Piñero, Inmaculada Cabezos, Alberto López-Navarro, Dolores de Fez, María T. Caballero, Vicente J. Camps

**Affiliations:** 1 Group of Optics and Visual Perception, Department of Optics, Pharmacology and Anatomy, University of Alicante, Crta San Vicente del Raspeig s/n 03016, San Vicente del Raspeig, Alicante, Spain; 2 Optometry Clinic, Prevention Service of the University of Alicante, Alicante, Spain

**Keywords:** Anterior chamber depth, Pachymetry, White-to-white, Irido-corneal angle, Scheimpflug imaging, VX120 system

## Abstract

**Background:**

To evaluate the intrasession repeatability of anterior chamber depth (ACD), central (CCT) and peripheral corneal thickness (PCT), white-to-white diameter (WTW), and irido-corneal angle (IA) measurements obtained with a multidiagnostic device in healthy eyes.

**Methods:**

A total of 107 eyes of 107 patients ranging in age from 23 to 65 years were examined with the VX120 system (Visionix-Luneau Technologies). Three consecutive measurements were obtained with this device to assess the intrasession repeatability of ACD, CCT, PCT at different nasal and temporal locations, WTW, and nasal and temporal IA. Data analysis included the calculation of within-subject standard deviation (S_w_), intrasubject precision (1.96xS_w_), coefficient of variation (CV) and intraclass correlation coefficient (ICC).

**Results:**

The S_w_ and CV for ACD was 0.03 mm and 1.16%, respectively, with an ICC of 0.992. The S_w_ values for central and peripheral pachymetric measurements were below 9 μm, with CV of less than 1.6% and ICC of 0.976 or higher. For IA measurements, S_w_ values of 0.84 or lower were found, with a CV between 1 and 2%, and an ICC of more than 0.970. The S_w_ for WTW was 0.24 mm and the CV was 1.95%. No statistically significant correlations were found between any anatomical parameter evaluated and their S_w_ and CV values associated (−0.220 ≤ *r* ≤ 0.204, *p* ≥ 0.125).

**Conclusions:**

The VX120 system is able to provide repeatable measurements of anatomical parameters in healthy eyes. Inter-observer repeatability should be evaluated in future studies.

## Background

Accurate measurement of different anatomical dimensions of the anterior segment is crucial in the eye care clinical practice [1]. These accurate measurements allow the clinician to perform a precise planning of refractive and intraocular surgical procedures, to check the viability of a specific type of surgical technique, and to perform comprehensive monitoring of ocular diseases. Thus, clinical decisions based on unreliable or not consistent data are avoided. There are several studies evaluating the repeatability of anterior segment anatomical measurements provided by different types of instruments, most of them based on Scheimpflug imaging or partial coherence interferometry [2–19]. Specifically, good intrasession repeatability data have been reported with a variety of devices for anterior chamber depth (ACD), [2–19] central corneal thickness (CCT), [4, 6, 10, 13–18] peripheral corneal thickness (PCT) [21], white-to-white corneal diameter, [3, 5, 8–10, 14, 15, 20] and irido-corneal angle (IA) [6, 10, 19, 21]. All these studies confirm the good performance of currently available devices for measuring the anatomical dimensions of the anterior segment of the eye.

Recently, a new multidiagnostic platform has been developed that provides automatic measurements of corneal topography, corneal, internal and ocular aberrations, pachymetry, anterior chamber depth, irido-corneal angle, pupil diameter under different luminance conditions and intraocular pressure (IOP), which is the VX120 system (Visionix-Luneau Technologies, Chartres, France). To date, there are no scientific studies evaluating the reliability of this device. The aim of the current study was to evaluate the intrasession repeatability of anterior segment anatomical measurements obtained with the VX120 system in a sample of normal healthy eyes.

## Methods

### Patients

A total of 107 healthy eyes of 107 patients ranging in age from 23 to 65 years old were enrolled in this prospective study of evaluation of a technology. All subjects were selected randomly from patients attending to the Optometry Clinic of the University of Alicante, where this investigation was developed. One eye only from each subject was chosen randomly for the study using a random number sequence (dichotomic sequence, 0 and 1) in order to avoid the potential interference of the correlation that often exists between the two eyes of the same person. All patients were informed previously about the study and signed an informed consent in accordance with the tenets of the Helsinki Declaration. An approval for the performance of the study was obtained from the Ethics Committee of the University of Alicante (Spain).

The inclusion criteria were the following: eyes without active ocular pathology, age of more than 18 years, and the presence of a refractive error between +5.00 and −10.00 D. The following conditions were defined as exclusion criteria for the study: any systemic pathology at the moment of examination, previous ocular surgery, glaucoma, less than 18 complete consecutive Placido rings projected on the cornea and therefore considered for the corneal analysis, pseudophakia, corneal ectatic diseases, and any other type of pathological condition of the eye.

### Examination protocol

**Fig. 1:**
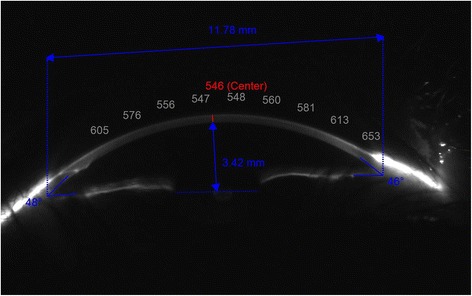
Horizontal scan obtained with the Scheimpflug camera of the VX120 system in which peripheral pachymetric measurements are done

A complete eye exam was performed in all cases that included measurement of uncorrected (UDVA) and corrected distance visual acuity (CDVA), manifest refraction, air tonometry (VX120 system, Visionix-Luneau Technologies, Chartres, France), and corneal topographic and anterior segment analysis with the VX120 system (Visionix-Luneau Technologies, Chartres, France). All measurements were performed by the same experienced examiner (ALN), taking three consecutive measurements in order to analyze the intrasession repeatability of some anatomical measurements. Specifically, the repeatability of the following parameters was evaluated: anterior chamber depth (ACD), peripheral corneal thickness at 1, 2 and 3 mm from vertex nasally (PCT_N1_, PCT_N2_, and PCT_N3_), peripheral corneal thickness at 1, 2 and 3 mm from vertex temporally (PCT_T1_, PCT_T2_, and PCT_T3_), central corneal thickness (CCT) (Fig. 1), nasal and temporal irido-corneal angles (IA_N_ and IA_T_), and horizontal white-to-white corneal diameter (WTW).

### The VX120 system

The VX120 system is a multidiagnostic platform that combines several technologies in order to provide an integral exam of the anterior segment of the eye. Specifically, the system combines a Scheimpflug imaging-based system, a Placido disk corneal topographer, a Hartmann-Shack aberrometer, and an air tonometer. The information used to provide all corneal topographic information is provided by a Placido disk system that projects 24 rings on the corneal surface, measuring more than 100,000 points. Refractive and ocular aberrometric data are obtained with a Hartmann-Shack system that measures 1500 points in 0.2 s in an area ranging from 2.0 to 7.0 mm of diameter. The Scheimpflug imaging-based system uses monochromatic blue light of 455 nm to obtain pachymetric measurements with a resolution of ± 1 μm, and iridocorneal angle measurements with a resolution of ± 1°. With the data obtained with all these technologies, the system also provides tangential and axial curvature data of the anterior corneal surface, internal wavefront aberrations, visual quality simulations, central and peripheral corneal pachymetry data, and IOP measurements.

### Statistical analysis

The statistical analysis was performed using the software SPSS version 15.0 for Windows (SPSS, Chicago, Illinois, USA). Normality of all data distributions was confirmed by means of the Kolmogorov-Smirnov test. Then, parametric statistics was applied. Intrasession repeatability for anatomical parameters was assessed according to the following variables: the within-subject standard deviation (S_w_) of the 3 consecutive measurements, intrasubject precision (1.96 x S_w_), and the intraclass correlation coefficient (ICC). The within-subject standard deviation (S_w_) is a simple way of estimating the size of the measurement error. The intraobserver precision was defined as (±1.96 x S_w_) and this parameter indicates how large is the range of error of the repeated measurements for 95% of observations. Finally, the ICC is an ANOVA-based type of correlation that measures the relative homogeneity within groups (between the repeated measurements) in ratio to the total variation. The ICC will approach 1.0 when there is no variance within repeated measurements, indicating total variation in measurements is due solely to variability in the parameter being measured. Furthermore, Pearson correlation coefficients were used to assess the correlation between different parameters evaluated. All statistical tests were 2-tailed, and *p*-values less than 0.05 were considered statistically significant.

## Results

Table 1 summarizes the main characteristics of the sample evaluated. The sample was comprised of 49 males (45.8%) and 58 females (54.2%), with a mean age of 47.8 years old. Table 2 summarizes the outcomes of the intrasession repeatability analysis for all anatomical parameters evaluated. The S_w_ and CV for ACD was 0.03 mm and 1.16%, respectively, with an ICC 0.992. The S_w_ values for central and peripheral pachymetric measurements were below 9 μm, with CV of less than 1.6% and ICC of 0.976 or higher. For IA measurements, S_w_ values of 0.84 or lower were found, with a CV between 1 and 2%, and ICC of more than 0.970. The S_w_ for WTW was 0.24 mm and the CV was 1.95%.

**Table 1:** Main characteristics of the sample evaluated

	Mean (SD)	Median	Range
Age (years)	47.8 (8.3)	47.0	23.0 to 65.0
Mean central corneal radius (mm)	7.92 (0.30)	7.86	7.26 to 8.84
Mean central corneal astigmatism (D)	0.82 (0.59)	0.75	0.00 to 3.75
Manifest sphere (D)	−0.43 (2.02)	0.00	−7.00 to 6.00
Manifest cylinder (D)	−0.40 (0.75)	0.00	−4.00 to 0.00
Manifest spherical equivalent (D)	−0.63 (2.03)	0.00	−7.33 to 5.50
Corneal eccentricity	0.34 (0.31)	0.43	−1.16 to 0.83

Abbreviations: *SD* standard deviation, *D* diopter

**Table 2:** Summary of the intrasession repeatability outcomes for the anatomical measurements evaluated with the VX120 system

	Overall mean (SD) Overall median (Range)	S_w_	Pr	CV (%)	ICC (Range 95% CI)
ACD (mm)	2.86 (0.29) 2.83 (2.10 to 3.77)	0.03	0.06	1.16	0.992 (0.989 to 0.994)
PCT_N3_ (μm)	592.04 (39.87) 601.00 (533.67 to 699.00)	8.98	17.59	1.52	0.976 (0.957 to 0.987)
PCT_N2_ (μm)	567.77 (37.83) 575.67 (506.33 to 675.33)	7.05	13.81	1.25	0.984 (0.971 to 0.991)
PCT_N1_ (μm)	574.89 (45.35) 549.67 (508.33 to 699.00)	7.70	15.09	1.34	0.984 (0.972 to 0.992)
CCT (μm)	545.88 (34.70) 544.67 (428.00 to 657.67)	6.41	12.56	1.17	0.983 (0.977 to 0.988)
PCT_T1_ (μm)	553.27 (37.44) 545.67 (492.00 to 664.33)	5.91	11.59	1.08	0.987 (0.977 to 0.993)
PCT_T2_ (μm)	568.70 (37.58) 573.33 (510.67 to 681.33)	6.06	11.87	1.07	0.987 (0.977 to 0.993)
PCT_T3_ (μm)	593.76 (38.58) 598.67 (529.00 to 708.00)	8.33	16.33	1.41	0.979 (0.963 to 0.989)
IA_N_ (°)	37.17 (4.35) 37.00 (28.33 to 46.00)	0.84	1.65	2.23	0.970 (0.946 to 0.984)
IA_T_ (°)	39.63 (4.59) 39.67 (30.67 to 50.33)	0.52	1.02	1.32	0.994 (0.988 to 0.997)
WTW (mm)	12.21 (0.57) 12.17 (10.99 to 13.94)	0.24	0.46	1.95	0.873 (0.823 to 0.911)

Abbreviations: *SD* standard deviation, *CV* coefficient of variation, *S*_*w*_ within-subject standard deviation, *Pr* intrasubject precision: 1.96 x S_w_, *ICC* intraclass correlation coefficient, *ACD* anterior chamber depth, *PCT*_*N1*_ *, PCT*_*N2*_ *, and PCT*_*N3*_ peripheral corneal thickness at 1, 2 and 3 mm from vertex nasally, *PCT*_*T1*_ *, PCT*_*T2*_ *, and PCT*_*T3*_ peripheral corneal thickness at 1, 2 and 3 mm from vertex temporally, *CCT* central corneal thickness, *IA*_*N*_ *and IA*_*T*_ nasal and temporal irido-corneal angles and *WTW* horizontal white-to-white corneal diameter

Table 3 displays the coefficients of correlation of the magnitude of different anatomical parameters and the S_w_ and CV values associated to each parameter. As shown, no statistically significant correlations were found (−0.220 ≤ *r* ≤ 0.204, *p* ≥ 0.125). Likewise, no significant correlations were found between mean keratometry, corneal eccentricity, corneal astigmatism or spherical equivalent and the S_w_ values obtained for each anatomical parameter evaluated (−0.253 ≤ *r* ≤ 0.275, *p* ≥ 0.125).

**Table 3:** Summary of correlations between the different anatomical parameters and their within-subject standard deviation (S_w_,) and coefficient of variation (CV) associated

	S_w_		CV	
	Pearson correlation coefficient	*p*-value	Pearson correlation coefficient	*p*-value
ACD (mm)	0.033	0.734	−0.058	0.550
PCT_N3_ (μm)	0.024	0.893	−0.082	0.652
PCT_N2_ (μm)	−0.125	0.487	−0.220	0.218
PCT_N1_ (μm)	0.101	0.577	0.000	0.999
CCT (μm)	0.149	0.127	0.056	0.565
PCT_T1_ (μm)	−0.069	0.704	−0.158	0.381
PCT_T2_ (μm)	−0.059	0.746	−0.142	0.431
PCT_T3_ (μm)	0.000	0.997	−0.100	0.580
IA_N_ (°)	0.204	0.254	0.122	0.498
IA_T_ (°)	0.039	0.827	−0.102	0.572
WTW (mm)	−0.109	0.282	−0.154	0.125

Abbreviations: *SD* standard deviation, *CV* coefficient of variation, *S*_*w*_ within-subject standard deviation, *Pr* intrasubject precision: 1.96 x S_w_, *ICC* intraclass correlation coefficient, *ACD* anterior chamber depth, *PCT*_*N1*_ *, PCT*_*N2*_ *, and PCT*_*N3*_ peripheral corneal thickness at 1, 2 and 3 mm from vertex nasally, *PCT*_*T1*_ *, PCT*_*T2*_ *, and PCT*_*T3*_ peripheral corneal thickness at 1, 2 and 3 mm from vertex temporally, *CCT* central corneal thickness, *IA*_*N*_ *and IA*_*T*_ nasal and temporal irido-corneal angles, and WTW horizontal white-to-white corneal diameter

## Discussion

The concept of integrating several clinical tests in one device in order to simplify the activity in clinical setting has been investigated extensively in the last years. Some multidiagnostic platforms combining different technologies have been developed and commercially released [1, 21–23]. One of the potential concerns about these systems if they are able to provide consistent and reliable measurements that can be considered as interchangeable with those measurements provided by gold-standard or widely tested devices. The current study was aimed at confirming if one experienced operator was able to obtain repeatable measurements of different anatomical parameters of the anterior segment with the multidiagnostic platform VX120. If this intrasession repeatability is confirmed, future studies will be conducted to analyze the inter-observer repeatability and the interchangeability of VX120 measurements with those obtained by other different currently available topography systems or tomographers.

In our study, the intrasession repeatability of ACD measurements was excellent, with S_w_ of 0.03 mm, CV of 1.16% and ICC of 0.992. This confirms that the multidiagnostic device evaluated is able to provide repeatable consecutive measurements of the depth of the anterior chamber of the healthy eye. Our intrasession repeatability outcomes for ACD are consistent with those reported with other commercially available devices, including Scheimpflug-based topography systems, [3, 5, 6, 10–14, 16–19] biometers, [2–4, 8, 9, 15, 17] and optical coherence tomographers [3, 11] (Table 4). Kurian et al. [2] obtained in 100 eyes evaluated with the IOL-Master (Carl Zeiss Meditec) and Lenstar 900 (Haag-Streit) optical biometry systems, S_w_ values for ACD of 0.04 and 0.06 mm, and CV of 1.22 and 1.99%, respectively. Kunert and coauthors [4] found a S_w_ value for ACD of 0.0098 mm with the optical biometry system IOL Master 700 (Carl Zeiss Meditec). Other authors have also reported S_w_ values below 0.06 mm for the same and other optical biometers [8, 9, 15, 17]. Likewise, several studies have evaluated the consistency of ACD measurements using Scheimpflug imaging-based topography systems, such as the Sirius (CSO), Galilei (Ziemer) and Pentacam (Oculus) systems. Shin et al. [5] reported an ICC > 0.980 for ACD measurements obtained with the Galilei G6 system in a sample of 140 eyes with cataract, and Savini et al. [19] obtained CV < 3.5% and ICC > 0.94 in 45 healthy eyes using the same system. With the Sirius system from CSO, Prakash et al. [6] obtained a mean value of 0.06 mm for 1.96xS_w_ corresponding to ACD measurements in a sample of 100 healthy subjects. Likewise, Masoud et al. [10] obtained a CV < 2% in 100 healthy eyes of 50 patients, Nasser et al. [16] a CV of 0.56% in 45 healthy eyes, and Montalbán et al. [18] S_w_, CV and ICC of 0.02 mm, 0.54% and 0.999 in 117 healthy eyes, respectively. Hernández-Camarena and coauthors [12] performed a comparative study of the repeatability of ACD measurements obtained with the Galilei G2 (Ziemer), Pentacam HR (Oculus) and Sirius (CSO) systems in 84 healthy eyes and found CV values below 1% in all cases. Shetty and coauthors [13] performed a similar comparison but in 55 keratoconus eyes and obtained S_w_ values of 0.03, 0.05 and 0.03 mm with the Pentacam, Galilei and Sirius systems, respectively. Therefore, the VX120 platform that uses Scheimpflug imaging for measuring ACD provides repeatable measurements of this anatomical parameter, with levels of reliability that are consistent with those found for other systems also based on Scheimpflug imaging or optical biometry (Table 4).

**Table 4:** Summary of the results of previous studies evaluating the consistency of anterior chamber depth (ACD), peripheral corneal thickness (PCT), central corneal thickness (CCT), iridocorneal angle (IA), and white-to-white corneal diameter (WTW)

Authors	Year	Number of eyes	Type of eyes	System	Parameter	1.96 Sw	Sw	ICC	CoV
*Kurian* et al. [2]	2016	100	Healthy	IOL Master 700 Lenstar 900	ACD	–	0.04 mm 0.06 mm	–	1.22% 1.99%
*Shajari* et al. [3]	2016	40	Healthy	Pentacam HR IOL Master 500 Lenstar 900 Visante OCT Pentacam HR IOL Master 500 Lenstar 900 Visante OCT	WTW ACD	0.5 0.5 0.5 0.6 0.3 0.3 0.3 0.3	–	–	–
*Shin* et al. [5]	2016	140	Cataract	Galilei G6	WTW ACD	–	–	> 0.980	–
*Kunert* et al. [4]	2016	120	Cataract	IOL Master 700	CCT ACD	–	19.5 μm 9.8 μm	–	–
*Prakash* et al. [6]	2016	100	Healthy	Sirius	CCT ACD IA	5 μm 0.06 mm < 2°	–	–	–
*Huang* et al. [8]	2015	52 46	Healthy Cataract	Aladdin	WTW ACD	–	0.80 mm	> 0.94 0.795	< 0.89%
*Srivannaboon* et al. [9]	2015	100	Cataract	IOL Master 500/700	WTW ACD	–	–	0.93 to 1.00	–
*Masoud* et al. [10]	2015	100	Healthy	Sirius	IA ACD	–	–	–	<2%
*Wang* et al. [11]	2015	71	Healthy	Galilei G2 Visante OCT Sirius Pentacam	ACD	–	0.04 to 0.07 mm	–	–
*Shetty* et al. [13]	2014	55	Keratoconus	Pentacam Galilei Sirius	ACD	–	0.03 mm 0.05 mm 0.03 mm	–	1.1% 1.3% 1.0%
*Hernández-Camarena* et al. [12]	2014	84	Healthy	Galilei G2 Pentacam HR Sirius	CCT ACD	–	–	–	< 1%
*Zhao* et al. [15]	2013	56	Healthy	Lenstar 900	WTW ACD CCT	–	0.274 mm 0.052 mm 14.24 μm	–	–
*Montalban* et al. [14]	2013	61	Keratoconus	Sirius	WTW CCT ACD	--- ---	0.07 mm 2.30 μm 0.02 mm	0.989 0.998 0.998	0.56% 0.51% 0.64%
*Chen* et al. [17]	2012	40	Healthy	Sirius Lenstar 900 Sirius Lenstar 900 Sirius Lenstar 900	WTW ACD CCT	–	0.04 mm 0.05 mm 0.02 mm 0.02 mm 3.10 μm 3.32 μm	–	–
*Montalban* et al. [18]	2012	117	Healthy	Sirius	WTW ACD CCT	–	0.06 mm 0.02 mm 2.80 μm	0.974 0.999 0.997	0.48% 0.54% 0.52%
*Nasser* et al. [16]	2012	45	Healthy	Sirius	ACD	–	–	–	0.56%
*Savini* et al. [19]	2011	45	Healthy	Galilei	CCT IA (4 quadrants) WTW	–	–	> 0.94	< 3.5%

Abbreviations: *SD* standard deviation, *S*_*w*_ within-subject standard deviation, *ICC* intraclass correlation coefficient, *CoV* coefficient of variation, *D* diopter

The intrasession repeatability of central and peripheral pachymetric readings obtained with the VX120 system in our sample was also excellent, with S_w_ values below 9 μm, CV below 1.6% and ICC of more than 0.97. Specifically, S_w_ for central corneal thickness was 6.41 μm in our sample, a value which is consistent with those reported using the Sirius system in healthy (Prakash et al. [6] S_w_: 5 μm; Montalbán et al. [18] S_w_: 2.80 μm; Chen et al. [17] S_w_: 3.10 μm) and keratoconus eyes (Montalbán et al. [14] S_w_: 2.30 μm). However, VX120 pachymetric repeatability outcomes were better than those reported for optical biometry systems (Zhao et al. [15] S_w_: 14.24 μm, healthy eyes; Kunert et al. [4] S_w_: 19.5 μm) (Table 4). Concerning peripheral pachymetry, the readings obtained with the VX120 system were very repeatable, with S_w_ values ranging from 5.91 to 8.98 μm. Milla and coauthors [20] evaluated the intrasession consistency of peripheral pachymetry obtained with the Scheimpflug imaging-based topography system Sirius from CSO and obtained S_w_ values ranging from 3.1 to 5.8 μm. These values are consistent with those found in the current study. Likewise, other authors have reported similar S_w_ or even worse than those obtained in the current series for minimal corneal thickness (Shetty et al. [13] S_w_ 9.33 μm Pentacam, S_w_ 11.64 μm Galilei, S_w_ 8.88 μm Sirius, Montalbán et al. [14] S_w_ 3.18 μm) (Table 4).

Besides ACD and pachymetry, the intrasession repeatability of WTW and iridocorneal angle measurements was also evaluated. In our series, excellent intrasession repeatability was found for WTW, with values of 0.24 mm, 1.95% and 0.873 for S_w_, CV and ICC, respectively. Shajari and coauthors [3] reported in a sample of 40 healthy subjects in which WTW was measured with the Pentacam HR, IOL-Master 500, Lenstar 900 and Visante systems, a value of approximately 0.5 μm for 1.96xS_w_. Likewise, excellent intrasession repeatability for WTW has been also reported by other authors using a variety of devices: Galilei (Shin et al. [5] ICC > 0.980 for cataract eyes; Savini et al. [19] ICC > 0.94 for healthy eyes), Lenstar 900 (Zhao et al. [15] Sw: 0.274 μm for healthy eyes), Aladdin biometer (Huang et al. [8] ICC > 0.94 for healthy eyes, ICC: 0.795 for cataract eyes), Sirius (Montalbán et al. [14] S_w_: 0.07 mm for keratoconus eyes, Montalbán et al. [18] S_w_: 0.06 mm for healthy eyes), and IOL-Master system (Srivannaboon et al. [9] ICC > 0.93 for cataract eyes). Concerning iridocorneal angle, an excellent intrasession repeatability was also obtained for nasal and temporal measurements over a horizontal scan, with Sw < 0.9°, CV < 2.3% and ICC > 0.97. This level of repeatability is consistent with that reported for other Scheimpflug imaging-based topography systems [6, 10, 19]. Prakash et al. [6] reported values of 1.96xS_w_ < 2° in iridocorneal angle measurements obtained with the Sirius system from CSO in a sample of 100 healthy subjects. Masoud et al. [10] found CV values below 2% for anterior chamber angle measurements obtained with the same topography system in another sample of 100 healthy eyes. Likewise, Savini et al. [19] found CV values below 3.5% and ICC values of more than 0.94 for iridocorneal angle measurements in four quadrants obtained using the Galilei system in 45 healthy eyes. Therefore, the multidiagnostic platform VX120 is also able to provide repeatable measurements of WTW and iridocorneal angle, with levels of intrasession repeatability comparable to those reported for other commercially available systems (Table 4).

Finally, the level of correlation between the magnitude of all anatomical variables evaluated and their S_w_ and CV values associated. Thus, we investigated if the level of repeatability of each anatomical parameter was dependent on its magnitude. This analysis revealed that this dependency was not present for any anatomical variable evaluated. Therefore, the VX120 system provides anatomical measurements of the anterior segment, with minimal repeatability errors that do not seem to increase in cases with extreme values within the normal range. More studies are needed to corroborate this finding in eyes with abnormal or pathological anterior segment, such as microcorneas and ectatic corneas.

The results of this study have only demonstrated that the VX120 system is able to provide repeatable measurements, which is crucial for being used as a diagnostic tool in clinical practice. However, future studies are necessary to evaluate the inter-observer repeatability and the interchangeability of the measurements provided by the VX120 platform with those obtained with other commercially available systems. In any case, good inter-observer repeatability is expected to be found considering that measurements are taken automatically by the VX120 system, with minimal intervention from the observer. Indeed, the observer does not have to focus or center because this is done automatically by the system.

## Conclusion

The multidiagnostic system VX120 seems to be able to provide consistent repeated measurements of ACD, central and peripheral pachymetry, WTW and iridocorneal angle in healthy eyes. The level of repeatability of the measurement of these anatomical parameters with the VX120 system is not dependent on the magnitude of such parameters, with the same precision ability for short and long eyes, small and large corneas, and eyes with deep and shallow anterior chamber within the normal range. Future studies should be conducted to confirm if this level of intrasession repeatability for the anatomical parameters evaluated is also observed in pathological eyes or after specific types of eye surgery. Furthermore, although measurements are done automatically by the device with minimal intervention of the operator, future inter-observer repeatability studies should be also performed.

## Abbreviations

ACD: Anterior chamber depth
CCT: Central corneal thickness
CDVA: Corrected distance visual acuity
CV: Coefficient of variation
IA: Irido-corneal angle
ICC: Intraclass correlation coefficient
IOP: Intraocular pressure
PCT: Peripheral corneal thickness
PCT_N1_, PCT_N2_, and PCT_N3_: Peripheral corneal thickness at 1, 2 and 3 mm from vertex nasally
PCT_T1_, PCT_T2_, and PCT_T3_: Peripheral corneal thickness at 1, 2 and 3 mm from vertex temporally
UDVA: Uncorrected distance visual acuity
WTW: White-to-white diameter
